# Some Clinical Notes on Membranous Enteritis

**Published:** 1895-03

**Authors:** C. Percival Crouch


### SOME CLINICAL NOTES ON MEMBRANOUS ENTERITIS.

BY

C. Percival Crouch, M.B. Lond., F.R.C.S. Eng.

---

I propose in this paper to give a short clinical history of a few cases of membranous enteritis that have come under my care during the last few years.

Sincerely do I wish that my limited experience enabled me to advocate any form of treatment likely to benefit those suffering from this very intractable complaint; but after trying many and various drugs by mouth, and medicated enemata by the bowel, I am most unwillingly bound to confess that as yet I have not found any remedy effect a permanent cure. In some few cases the patient improved for a time while under treatment, but as in almost every instance the trouble appeared again after a very short interval, and as, moreover, the patients had experienced this same freedom from symptom without undergoing any treatment at all, I was forced to the conclusion that we have not yet discovered any effectual treatment for this disease.

Mrs. M., aged 60. A very thin, ill-nourished woman, of melancholy temperament. She has always had wretched health, and at the present time has marked signs of pulmonary phthisis. For many years she has suffered, at short intervals, from attacks of acute dyspepsia, accompanied by very obstinate constipation; and at these times she experiences great pain in the abdomen.

When I was called in to see her, I found her suffering great pain in the abdomen. She was sitting up in bed rocking to and fro, and pressing her abdomen as firmly as she could against her thighs, as in that position she obtained most relief from the pain. She was also in great mental distress, as she was fully convinced that the membranes she was passing in great quantity were the "lining walls of her bowels," and I doubt whether, in spite of my earnest assurance, she was at all convinced to the contrary. She was looking miserably ill, and was moaning with pain.

The abdomen was not at all distended, and I found nothing abnormal present. Pressure relieved the pain, and there was but little tenderness. When the bowels acted the pain was much increased, and was followed by great straining after the membranes had been ejected. A little bright blood was passed, the source of which was doubtful. She did not suffer from piles.

On examining the motions they were found to consist of hard round fæcal lumps, with strips of white membrane wrapped round them. There were also present bits of membrane of various sizes, the largest measuring about six or seven inches long by three to four inches wide. The membrane itself presented a firm opaque-white structure, like an hydatid cyst-wall. These membranes dissolved in water after being left standing in it for some hours, leaving the water slightly turbid. Besides the membranes, the patient passed at times big lumps of mucus in semi-solid masses.

After a week or ten days the symptom disappeared, and she returned to her usual weakly state of health.

The treatment of this case was at first limited to relief of pain by opium, which I was obliged to give in small doses to enable her to obtain ease, and relieve pain caused by eating. Other drugs by mouth failed to do any good at all.

Mrs. R., aged 47. An energetic woman, of active habits; fairly well nourished; naturally of a bright disposition. She has suffered from obstinate constipation for many years; and from time to time has passed mucus by the bowel, with hard lumps of fæcal matter. For six months she has been more or less confined to her bed through a serious injury to her knee, and during this period she has passed mucus more frequently and has suffered from prolonged attacks of acute dyspepsia.

At the present time (July, 1892) she has symptoms of extreme tenderness of the mucous membrane of most of the alimentary canal. On swallowing food at all hot she experiences pain all down the œsophagus, which is worst at the cardiac orifice as the food enters the stomach. The pain in the stomach is almost constant, and there is great tenderness over it at the epigastrium on pressure. The pain in the abdomen is also almost constant, and is so severe as to keep her awake at night. When the bowels are moved the pain is much aggravated, and seems to pass along the line of the colon down the rectum. At such times too there is some tenderness in the region of the colon. There is no particular distension of the abdomen; and nothing abnormal can be felt in it either by external palpation or *per rectum*.

To obtain some relief from the pain, she sat up in bed with the knees up to her chin, pressing the abdomen against her thighs. This attack lasted six weeks, and during the whole of that time there was most obstinate constipation, so that enemas and purgatives were always required in order to obtain any action at all. For more than a fortnight there was so much pain on eating, accompanied by vomiting, that nutrient suppositories by rectum had to be administered, while only a very limited supply of food by mouth was given. Hot food, spirits or champagne could not be taken, as they caused so much burning pain immediately on being swallowed.

During this time I was compelled to give her small doses of opium in pill form, some fifteen minutes before food, to act as a local sedative to the stomach, as otherwise the taking of even the smallest amount of food was promptly followed by severe pain and vomiting.

This attack lasted altogether about six weeks, when the symptoms began to improve very slowly—the pain went, the mucus grew less in amount, and the bowels began to act naturally again, so that for a time they acted regularly every day without any assistance being required from either purgatives or enemata.

Strange to say, the patient lost very little flesh during this illness; and neither her heart, lungs nor kidneys showed any signs of disease. I may mention that she had choroiditis in both eyes.

The treatment of this patient was undertaken very thoroughly—I may even say heroically, as she was a woman of great pluck and perseverance and followed up any line of treatment I suggested with great determination. In spite, however, of the best endeavour on the part of both doctor and patient, I am sorry to say that though at times I thought certain drugs produced good effect, still they all failed to do good after a short time, and the patient's condition remained *in statu quo*. By the mouth I gave nitrate of silver pills, potassium iodide, arsenic, and other remedies as charcoal, and various antiseptics as benzo-naphthol, without avail. By the rectum I administered, by enema, tannic acid, boracic acid, in solution; also a lotion of salicylate of bismuth and opium. In order to give the rectal lotion a chance of coming into contact with the mucous membrane, I adopted the plan of washing out the bowel very thoroughly with hot water before passing the lotion up. The patient lay in bed with the buttocks raised high by pillows under them. The hot water, to the extent of nearly two gallons, was then gently passed up through a tube from a vessel suspended some three feet above the patient. When the water was returned free from mucus, the medicated lotion was thrown up and left for some two or three hours, the patient remaining with buttocks raised during that time. This very irksome treatment was persevered in for over a fortnight, at first every day and then every other day, but failed to reduce the amount of mucus passed. On the morning following a thorough emptying of the bowels from mucus by this treatment, there was passed as much mucus as ever—on some days enough being passed to half-fill a big chamber-pot. Finding active treatment do no good, I finally treated the symptoms of pain and constipation, and after some weeks the patient slowly improved as stated above.

Soon after this attack she went elsewhere to live, and at my request, a few days ago, she wrote the following account of herself. Her general health has been better, and she suffers less from constipation. Every month however, and sometimes oftener, she has an attack of the same kind. There is always sickness, pain and tenderness. Abundant mucus is passed, sometimes tinged with blood. The motions are then passed in hard lumps. Any mental worry always brings on an attack.

The next case is almost identical with the last. I give it briefly.

Miss C., aged 40, July, 1894. An unhealthy-looking woman; fairly well nourished. Always subject to constipation and piles, and at times has acute attacks of dyspepsia. These attacks are always accompanied by great pain and tenderness in abdomen, and vomiting. At times she has brought up coffee-ground vomit, due to the presence of blood. At present she is suffering from very great pain in the abdomen, which is much swollen and tender to pressure. She is unable to wear her corsets, owing to the tenderness. Pain is worst along line of left colon. Eating brings on the pain at once, and is often followed by vomiting. Constipation is very troublesome. She is passing hard lumps of fæces and much mucus and membranes, with streaks of blood. She is using large quantities of hot water every day to obtain an action—as much as two or three pints at a time.

The treatment was directed to improving her general health, owing to very considerable indiscretion in her diet and general manner of living. I was enabled to improve her health; but the mucus continued to pass, and still continues to the present time, when she wrote a few days ago to tell me of her progress.

One more case I will mention, as being different from the others.

In this case the complaint occurred in a little girl of seven years of age while suffering from some slight ailment. The nurse told me one morning that the child was passing "worms." I examined the so-called "worms," and found they were membranes or casts from the bowel. These casts were passed on three or four occasions only, and during that time there was no constipation or diarrhœa. The child did not complain of any pain or tenderness in the abdomen at any time. Since then, now two years, she has not passed any more membranes, and her health has been good.

In discussing and analysing these cases, I would direct attention chiefly to the following points:

It is apparently a complaint which does not yield to treatment in the majority of cases. It may improve while the patient is under treatment, but it also improves for a time without any treatment at all.

It is not a fatal complaint. People may have it for years and pass vast quantities of mucus, and yet look fairly well at the end of that time.

It often improves temporarily, and then returns after a short or long interval. In some cases it recurs at regular times, and continues to do so every month or so for years.

It almost always occurs in dyspeptic and somewhat neurotic patients.

It is essentially a disease which is affected by the mental state of the patient. In some cases worry always brings on an attack, while freedom from care is almost essential to its cure.

